# Inactivation of antibiotic-resistant bacteria *Escherichia coli* by electroporation

**DOI:** 10.3389/fmicb.2024.1347000

**Published:** 2024-01-25

**Authors:** Saša Haberl Meglič, Dejan Slokar, Damijan Miklavčič

**Affiliations:** ^1^Faculty of Electrical Engineering, University of Ljubljana, Ljubljana, Slovenia; ^2^Centre of Excellence for Biosensors, Instrumentation and Process Control, Ajdovščina, Slovenia

**Keywords:** electroporation, antibiotic resistant bacteria, inactivation, tetracycline, chloramphenicol, *Escherichia coli*

## Abstract

**Introduction:**

In modern times, bacterial infections have become a growing problem in the medical community due to the emergence of antibiotic-resistant bacteria. In fact, the overuse and improper disposal of antibiotics have led to bacterial resistance and the presence of such bacteria in wastewater. Therefore, it is critical to develop effective strategies for dealing with antibiotic-resistant bacteria in wastewater. Electroporation has been found to be one of the most promising complementary techniques for bacterial inactivation because it is effective against a wide range of bacteria, is non-chemical and is highly optimizable. Many studies have demonstrated electroporation-assisted inactivation of bacteria, but rarely have clinical antibiotics or bacteria resistant to these antibiotics been used in the study. Therefore, the motivation for our study was to use a treatment regimen that combines antibiotics and electroporation to inactivate antibiotic-resistant bacteria.

**Methods:**

We separately combined two antibiotics (tetracycline and chloramphenicol) to which the bacteria are resistant (with a different resistance mode) and electric pulses. We used three different concentrations of antibiotics (40, 80 and 150 µg/ml for tetracycline and 100, 500 and 2000 µg/ml for chloramphenicol, respectively) and four different electric field strengths (5, 10, 15 and 20 kV/cm) for electroporation.

**Results and discussion:**

Our results show that electroporation effectively enhances the effect of antibiotics and inactivates antibiotic-resistant bacteria. The inactivation rate for tetracycline or chloramphenicol was found to be different and to increase with the strength of the pulsed electric field and/or the concentration of the antibiotic. In addition, we show that electroporation has a longer lasting effect (up to 24 hours), making bacteria vulnerable for a considerable time. The present work provides new insights into the use of electroporation to inactivate antibiotic-resistant bacteria in the aquatic environment.

## 1 Introduction

In the days before antibiotics, infectious diseases often reached epidemic proportions and cost millions of lives. The discovery of antibiotics to treat infectious diseases is one of the most important achievements in the history of medicine and has become one of the pillars of modern medicine (Friedman et al., 2016). Although antibiotics have long been a mainstay in the treatment of bacterial infections, their overuse and misuse (especially in livestock industry), combined with inadequate infection prevention, has led to increasing bacterial resistance (Levy and Marshall, 2004). The dramatic increase in antibiotic resistance therefore makes bacterial infectious diseases again a global threat and medical challenge. Globally, approximately 4.95 million deaths are associated with bacterial resistance to antibiotics in 2019 (Murray et al., 2022), and estimates suggest this number could increase to at least 10 million people per year by 2050 (de Kraker et al., 2016). The development of novel approaches with multiple defense strategies is therefore critical for efficient inactivation of drug-resistant bacteria. Electroporation, a procedure in which electric fields are applied to bacterial cells, has shown promise as an adjunct to antibiotic treatment (Steele et al., 1994; Novickij et al., 2018; Vadlamani et al., 2018; Rubin et al., 2019; Clemente et al., 2020; Kuyukina et al., 2020; Martens et al., 2020; Vadlamani et al., 2020; Lovsin et al., 2021).

Electroporation (under this name) was first described 50 years ago (Neumann and Rosenheck, 1972) and causes transient increase in permeability of the cell membrane by applying high-voltage electric field pulses. This technique has gained attention as an effective, non-chemical method for introducing molecules into cells, including foreign DNA (Potocnik et al., 2022; Rakoczy et al., 2022) and membrane-impermeable anticancer drugs (i.e., electrochemotherapy) (Yarmush et al., 2014). At stronger electric fields, cells are damaged, leading to cell death. Such application also known as irreversible electroporation has been previously used to inactivate bacteria in various environments (Saulis, 2010; Gomez et al., 2019; Zhou et al., 2023) and has recently been proposed as a non-thermal ablation method for cancer (Geboers et al., 2020) and cardiac tissue (Reddy et al., 2023; Verma et al., 2023). Several studies have reported that electroporation, in combination with other treatments (e.g., ultrasound, mild heat etc.), can significantly reduce bacterial populations also in liquid foods (Garner, 2019).

To combat the growing threat of bacterial resistance to antibiotics researchers have turned to new, combined treatments for inactivating bacteria, including combining antibiotics with electroporation (Steele et al., 1994; Novickij et al., 2018; Vadlamani et al., 2018; Rubin et al., 2019; Clemente et al., 2020; Kuyukina et al., 2020; Martens et al., 2020; Vadlamani et al., 2020; Lovsin et al., 2021). As early as the 1990s, it was observed that the recovery of electroporated bacteria was significantly reduced in the presence of tetracycline-based antibiotics (Steele et al., 1994). Fewer transformants were produced in the presence of tetracycline or a tetracycline-related antibiotic. The mechanism of lower transformation by tetracycline-related antibiotics was however not explained. It was not until 15 years later, that the next study showed the possibility of combining electric currents and antibiotics to inactivate bacteria (del Pozo et al., 2009), followed by a study in which the flow of direct current through the biofilm of *Staphylococcus aureus* increased the sensitivity of bacteria to gentamicin (Zhang et al., 2014). Electroporation was also shown to completely eradicate *Staphylococcus aureus* in the presence of subclinical inhibitory concentrations of the antibiotic (Korem et al., 2018). Later, it was suggested that synergistic bacterial inactivation by combining antibiotics and electroporation can be used to disinfect large areas of infected wounds (Rubin et al., 2019). Some studies also show that very short pulses (in the nanosecond range) in combination with antibiotics effectively inactivate bacteria (Vadlamani et al., 2018; Martens et al., 2020; Vadlamani et al., 2020). Electroporation can also be used as an adjuvant to inactivate antibiotic-resistant bacteria, although the rate of inactivation is highly dependent on the concentration of the antibiotic and/or the strength of the electroporation pulses (Novickij et al., 2018; Kuyukina et al., 2020).

Due to increasing antibiotic resistance, also alternative antimicrobials are being explored, some to replace existing antibiotics, others to complement them or to explore their potential in the food industry (Clemente et al., 2020). The inactivation rate of various bacteria in a buffer system was shown to increase by combining electroporation with alternative antimicrobial agents (nisin, lactic acid), with reductions of more than 5 log units (Munoz et al., 2012). These results prompted further interest in validating the procedures in real food matrices (Clemente et al., 2020). Many studies have highlighted the potential of electroporation in combination with antibacterial peptides (Ravensdale et al., 2016), cauliflower and mandarin byproduct infusions (Sanz-Puig et al., 2016), nisin-loaded nanoparticles (Novickij et al., 2016), lysine (Svediene et al., 2021), or acetic and formic acids (Novickij et al., 2019; Perminaite et al., 2023) to inactivate different bacteria.

Another problematic reservoir for antibiotic-resistant bacteria and also for antibiotics and antibiotic-resistance genes is wastewater from hospitals and livestock farms, due to the excessive use of antibiotics in these facilities (Avatsingh et al., 2023; Marutescu et al., 2023; Mehanni et al., 2023). Therefore, thorough surveillance of wastewater is essential to protect public health and the environment (Tiwari et al., 2022). Currently, various chemical or non-chemical water, wastewater, or sludge treatments are in use (Guo et al., 2013; Jiménez-Tototzintle et al., 2018; Zhang et al., 2020; Cuetero-Martínez et al., 2023), of which many are not very effective or have other environmental or mechanical drawbacks (Zhang et al., 2020; Wang et al., 2021). Electroporation has been shown to be highly versatile, reproducible, non-genotoxic (Gusbeth et al., 2009), and effective on a wide variety of microorganisms (Mosqueda-Melgar et al., 2008). Moreover, bacteria are unlikely to develop resistance to electroporation due to its physical mechanism of action (Gusbeth et al., 2009), as has been also demonstrated in mammalian (Polajžer and Miklavčič, 2020) and bacterial (Gusbeth et al., 2009) cells. Electroporation, which makes the bacterial membrane permeable and thus increases the uptake of antibiotics, has proven to be a promising complementary technique for the treatment of wastewater (Gusbeth et al., 2009; Lovsin et al., 2021). Understanding how antibiotics and electroporation inactivate bacteria is critical not only for developing effective strategies to treat bacteria but also to alleviate antibiotic resistance. Further research is also needed to optimize and standardize the conditions for electroporation to ensure its effectiveness.

Our study aims to contribute to this goal by combining a treatment regimen that involves the use of antibiotics and electroporation to inactivate antibiotic-resistant bacteria. In the study, we compared electroporation-potentiated bacterial inactivation of antibiotics (tetracycline and chloramphenicol) with similar modes of action (inhibition of bacterial protein synthesis) to which bacteria have different resistance mechanisms. The mechanism of tetracycline resistance is that the bacterium has an efflux pump and pumps tetracycline out of the cell. The mechanism of chloramphenicol resistance is that the bacterium alters the antibiotic (by acetylation), rendering it non-functional.

## 2 Materials and methods

### 2.1 Bacterial strain cultivation and sample preparation

**Figure 1:**
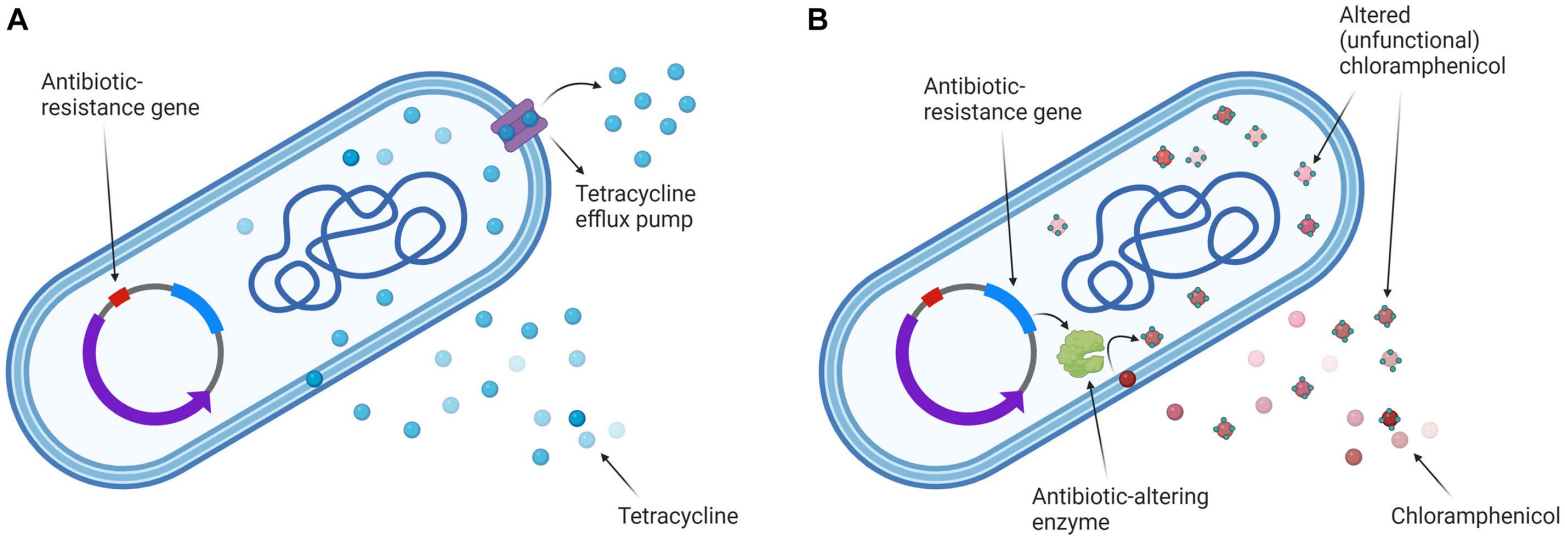
*Escherichia coli* K12 ER2420 mechanisms of tetracycline and chloramphenicol resistance. **(A)** The mechanism of tetracycline resistance: efflux pump is pumping tetracycline out of the cell; **(B)** The mechanism of chloramphenicol resistance: the bacterium alters the antibiotic (by acetylation), rendering it non-functional. Created with BioRender.com.

A strain of *Escherichia coli* K12 ER2420 containing the plasmid pACYC184 which carries a tetracycline and chloramphenicol resistance gene (New England BioLabs Inc., Ipswich, Massachusetts, USA), was used for the experiments (see Figure 1). Bacterial cells were grown overnight at 37°C with shaking (180 rpm) in Luria broth medium (Sigma-Aldrich Chemie GmbH, Schnelldorf, Germany) spiked with 40 μg/mL tetracycline (Sigma-Aldrich Chemie GmbH, Schnelldorf, Germany; #T3383) and 40 μg/mL chloramphenicol (Sigma-Aldrich Chemie GmbH, Schnelldorf, Germany; #C0378). The sample was then diluted with fresh Luria broth medium to which both antibiotics had been added and grown at 37°C with shaking (180 rpm) until an optical density (OD600) of approximately 0.400 was reached. At this density, the bacterial cells reached the early exponential phase. The cell pellet was then collected by centrifugation (4248 x *g*, 30 min, 4°C) and resuspended in 250 mM sucrose to minimize the effects of osmotic stress. The final conductivity of the sample was approximately 40 μS/cm.

### 2.2 Antibiotics preparation

Antibiotics to which the bacteria are resistant - tetracycline and chloramphenicol - were used. In fact, chloramphenicol is rarely used because of its known severe side effects (Oong and Tadi, 2023), so this antibiotic is only presented as a model in this study.

Both antibiotics were added to bacterial cells at different concentrations, and bacterial viability was determined (as described in Section 2.6.). To compare the effect of the two antibiotics on bacterial inactivation, antibiotic concentrations were chosen to achieve three different inactivation rates at which a reduction of approximately −0.060, −0.240, or − 0.930 log was achieved. These antibiotic concentrations were 40, 80 and 150 μg/mL for tetracycline and 100, 500 and 2000 μg/mL for chloramphenicol, respectively.

### 2.3 Incubation of bacterial cells after treatment and bacterial viability assessment

To mimic the treatment in nature, where the antibiotic is present in the water for a while, but then its concentration slowly decreases with the influx of a fresh stream, we first incubated the treated bacterial cells with the antibiotic and then plated them on the LB agar without antibiotic.

Therefore, immediately after treatment, a volume of 100 μL was taken from each sample and mixed with 100 μL of Luria broth medium (control sample and electroporated sample only) or 100 μL of Luria broth medium spiked with the antibiotic (samples treated with electroporation and antibiotic or antibiotic only) at the final concentration. The sample was then incubated for 3 h at 37°C with shaking, and a serial dilution of 20 μL sample aliquots in 0.9% NaCl was performed. Ten microliters of each dilution were plated on the LB agar without antibiotic and incubated at 37°C for 24 h. The colony forming units per ml (CFU/ml) were calculated from the bacterial counts. The reduction in bacterial cell count was expressed as log (N/N0), where N is the number of CFU/ml in the treated sample and N0 is the number of CFU/ml in the control sample.

### 2.4 Effect of electroporation on antibiotic activity

To exclude a negative effect of the electric pulses on antibiotic activity, the antibiotics alone were exposed to the electric pulses with the highest electric field strength (20 kV/cm) used in our study in a separate experiment. A suspension (140 μL) of both antibiotics at different concentrations (see Section 2.2) was placed between two stainless steel plate electrodes of rectangular shape (size of electrode area 0.6 × 2.8 cm) with a distance of 1 mm between the plates and exposed to a sequence of eight pulses with a duration of 100 μs, an electric field strength of 20 kV/cm and a repetition rate of 1 Hz.

The electric field strength was estimated as:


$$E=\frac{U}{d}$$

where *U* denotes the applied voltage and *d* the distance between the electrodes (*d* = 1 mm).

For the electroporation a square wave electric pulse generator HVP-VG (IGEA s.r.l., Carpi, Modena, Italy) was used. After treatment, the 100 μL of tetracycline (in the concentration of 40, 80 or 150 μg/mL) or chloramphenicol (in the concentration of 100, 500 or 2000 μg/mL) was added to the 100 μL of bacterial suspension (prepared as described in Section 2.1), whereupon the bacterial cells were incubated as described in Section 2.3. Bacterial suspensions that had not been exposed to electroporation or the antibiotic, but were also incubated as treated samples (see Section 2.3) served as controls.

### 2.5 Inactivation of *Escherichia coli* by antibiotics and electroporation

*Escherichia coli* cells resuspended in 250 mM sucrose (140 μL) were placed between two stainless steel plate electrodes of rectangular shape (size of electrode area 0.6 × 2.8 cm) with a distance of 1 mm between the plates and subjected to high voltage electric pulses at room temperature (22°C) using a square wave electric pulse generator HVP-VG (IGEA s.r.l., Carpi, Modena, Italy). Before the electroporation we added to each sample a different concentration of the antibiotic: (i) tetracycline: 0, 40, 80 or 150 μg/mL or (ii) chloramphenicol: 0, 100, 500 or 2000 μg/mL (see Section 2.2.).

A sequence of eight pulses, each with a duration of 100 μs, a pulse repetition rate of 1 Hz, and different electric field strengths (0, 5, 10, 15, and 20 kV/cm) was applied.

After treatment, bacterial cells were incubated as described in Section 2.3. Bacterial suspensions that had not been exposed to electroporation or the antibiotic, but were also incubated as treated samples (see Section 2.3) served as controls.

### 2.6 Inactivation of *Escherichia coli* by antibiotics and electroporation – Time dynamics

In order to study the dynamics of electroporation as a potentiator of antimicrobial efficacy, the antibiotics were added to bacterial cells (prepared as described in Section 2.1) at different times after electroporation. First, the bacterial cells were electroporated and then left at room temperature (22°C) for various periods of time (0 min, 1 min, 5 min, 10 min, 15 min, 30 min, 1 h, 2 h, 3 h, or 24 h). After specific time, an antibiotic was added to observe a possible synergistic effect.

To investigate the combined effect of electroporation and antibiotics (and a possible synergistic effect), a lower concentration of both antibiotics (tetracycline 40 μg/mL or chloramphenicol 500 μg/mL) was added and a lower electric field strength was applied (8 × 100 μs, 10 kV/cm, 1 Hz).

Bacterial cells were also treated with antibiotics or electroporation only and incubated for the same time (0 min, 1 min, 5 min, 10 min, 15 min, 30 min, 1 h, 2 h, 3 h, or 24 h) at room temperature (22°C).

After incubation (0 min, 1 min, 5 min, 10 min, 15 min, 30 min, 1 h, 2 h, 3 h, or 24 h), bacterial cells were further incubated as described in Section 2.3. Bacterial suspensions that had not been exposed to electroporation or the antibiotic, but were also incubated as treated samples (see Section 2.3) served as controls (each time point had its own control).

### 2.7 Statistical analysis

Experiments were repeated three times on three different days to demonstrate repeatability. Results were analysed using an unpaired *t* test (SigmaPlot 11.0, Systat Software, Richmond, CA) and considered statistically different at *p* < 0.05. Each bar or data point in the results represents the mean of the three experiments, with standard deviations shown as error bars.

## 3 Results

### 3.1 Effect of electroporation on antibiotic activity

Since in our study the antibiotics were added before the application of the electric pulse, it is reasonable to assume that electroporation could damage the antibiotic, rendering it ineffective. To confirm or refute the above hypothesis, in separate experiments the antibiotics were exposed to the electric pulses with the highest electric field strength used in our experiments (20 kV/cm) and then added to the bacterial cells.

**Figure 2:**
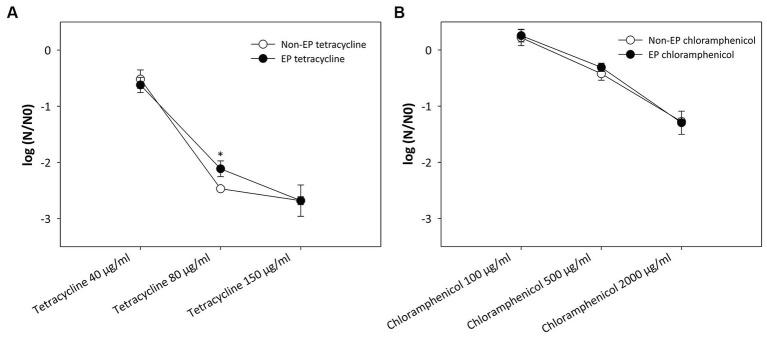
Effect of electroporation on the activity of **(A)** tetracycline and **(B)** chloramphenicol. A suspension of the two antibiotics at different concentrations was subjected to a sequence of eight pulses with a duration of 100 μs, an electric field strength of 20 kV/cm and a repetition rate of 1 Hz. Bacterial cells (*Escherichia coli* ER2420) were grown to early exponential phase, and the electroporated or non-electroporated antibiotic was added. Bacterial cell count reduction is expressed as log (N/N0), where N is the number of CFU/ml in the treated sample and N0 is the number of CFU/ml in the control sample. An asterisk (*) indicates a statistically significant (*p* < 0.05) difference compared to the addition of a non-electroporated antibiotic. Values represent means (all tests were performed in triplicate), and error bars were calculated using the standard deviation. The abbreviations “non-EP” or “EP” stands for “non-electroporated” or “electroporated” antibiotic.

The results presented in Figure 2 refute above hypothesis and show that electroporation has no effect on antibiotic activity. Both antibiotics retained their efficacy despite exposure to high-voltage electric pulses. A statistically significant (*p* = 0.013) difference between the electroporated and non-electroporated antibiotic was only observed when using 80 μg/mL tetracycline (Figure 2A).

### 3.2 Inactivation of *Escherichia coli* by antibiotics and electroporation

**Figure 3:**
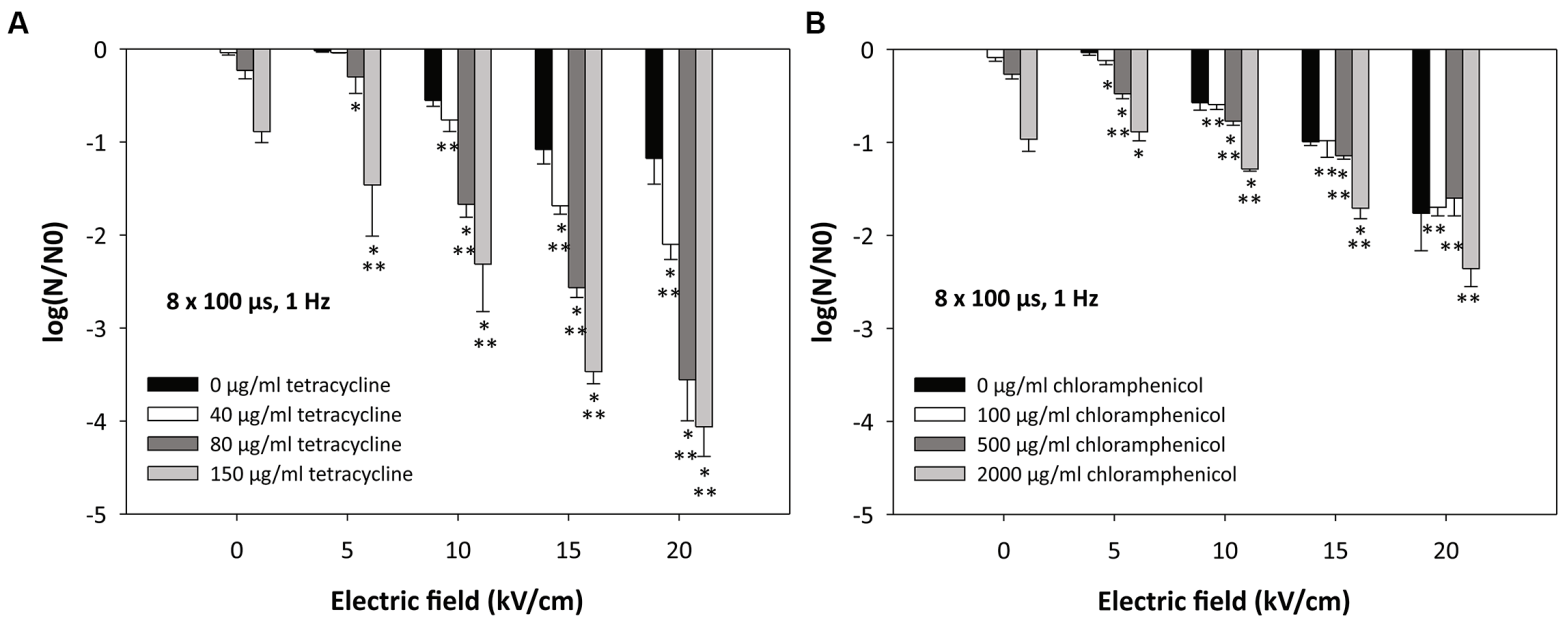
Inactivation of *Escherichia coli* by **(A)** tetracycline, **(B)** chloramphenicol, and electroporation. Bacterial cells (*Escherichia coli* ER2420) were grown to early exponential phase and were subjected to electroporation (black bars) or treatment with tetracycline or chloramphenicol alone or in combination with electroporation. The electroporation parameters were: 8 pulses of 100 μs duration, with a repetition rate of 1 Hz and different electric field strengths (5, 10, 15 and 20 kV/cm). The concentrations of added tetracycline were 40, 80, and 150 μg/mL (Figure 2A) and those of added chloramphenicol were 100, 500, and 2000 μg/mL (Figure 2B). Bacterial cells were treated at room temperature (22°C). Bacterial cell count reduction is expressed as log (N/N0), where N is the number of CFU/ml in the treated sample and N0 is the number of CFU/ml in the control sample. An asterisk (*) indicates a statistically significant (p < 0.05) difference from electroporation treatment alone, and two asterisks (**) indicate a statistically significant (*p* < 0.05) difference from antibiotic treatment alone. Values represent means (all tests were performed in triplicate), and error bars were calculated using the standard deviation.

*Escherichia coli* cells were treated with electric pulses (8 × 100 μs, 5–20 kV/cm, 1 Hz) or antibiotics alone and in combination with tetracycline (40, 80, or 150 μg/mL) (Figure 3A) or chloramphenicol (100, 500, or 2000 μg/mL) (Figure 3B). Bacterial viability was assessed in all single and combined treatments.

When *E. coli* cells were treated with electroporation alone (black bars in Figures 3A,B), a decrease in bacterial viability was observed with increasing pulse amplitude, i.e., with increasing electric field. The lowest electric field (5 kV/cm) had minimal effect on bacterial viability (−0.03 log reduction). As expected, the highest electric field (20 kV/cm) was most effective. At these conditions, a decrease in bacterial viability was observed (black bars in Figures 3A,B).

Figure 3 shows that the combination of antibiotics and electroporation can significantly improve the efficacy of inactivation compared with electroporation alone or the use of antibiotics alone. When only tetracycline was added to bacterial cells at increasing concentrations (see also Figure 2A) (without electroporation), bacterial cell survival decreased (Figure 3A; 0 kV/cm; see also Figure 2A). The log reductions for tetracycline concentrations of 40, 80, or 150 μg/mL were − 0.04 ± 0.02, −0.23 ± 0.09, and − 0.89 ± 0.12, respectively. The combination of both treatments (electroporation and tetracycline) resulted in additional loss of cell viability (Figure 3A). A statistically significant effect of combined treatment (compared to treatment with electroporation or tetracycline alone) was observed when 10 kV/cm was applied and 80 or 150 μg/mL of tetracycline was added. At higher electric fields (15 and 20 kV/cm), all concentrations of tetracycline administered resulted in an additive effect with electroporation. The strongest effect of the combined effect was observed at the highest electric field (20 kV/cm) and the highest tetracycline concentration (150 μg/mL), where the log reduction was −4.06 ± 0.32.

Figure 3 also shows that when only chloramphenicol was added to bacterial cells at increasing concentrations (see also Figure 2B) (without electroporation), bacterial cell survival decreased (Figure 3B; 0 kV/cm; see also Figure 2B). The log reductions for chloramphenicol concentrations of 100, 500, or 2000 μg/mL were − 0.09 ± 0.04, −0.27 ± 0.05, and − 0.97 ± 0.13, respectively. Additional loss of cell viability was observed with the combination of therapies (electroporation and chloramphenicol) (Figure 3B), although the loss of viability was not as high as with the combined treatment of tetracycline and electroporation. At the highest electric field strength (20 kV/cm), no statistically significant difference was found between the combined treatment and electroporation alone.

### 3.3 Inactivation of *Escherichia coli* by antibiotics and electroporation – time dynamics

In our study, we also investigated the dynamics of electroporation as a potentiating agent for antimicrobial activity. Antibiotics were added to bacterial cells at different times after electroporation. Bacterial cells resuspended in 250 mM sucrose were treated with electroporation (8 × 100 μs, 10 kV/cm, 1 Hz) and left at room temperature (22°C). Subsequently, antibiotic (tetracycline at a concentration of 40 μg/mL or chloramphenicol at a concentration of 500 μg/mL) was added at different time points (different incubation time) after electroporation (0 min-immediately after electroporation, 1 min, 5 min, 10 min, 15 min, 30 min, 1 h, 2 h, 3 h or 24 h).

**Figure 4:**
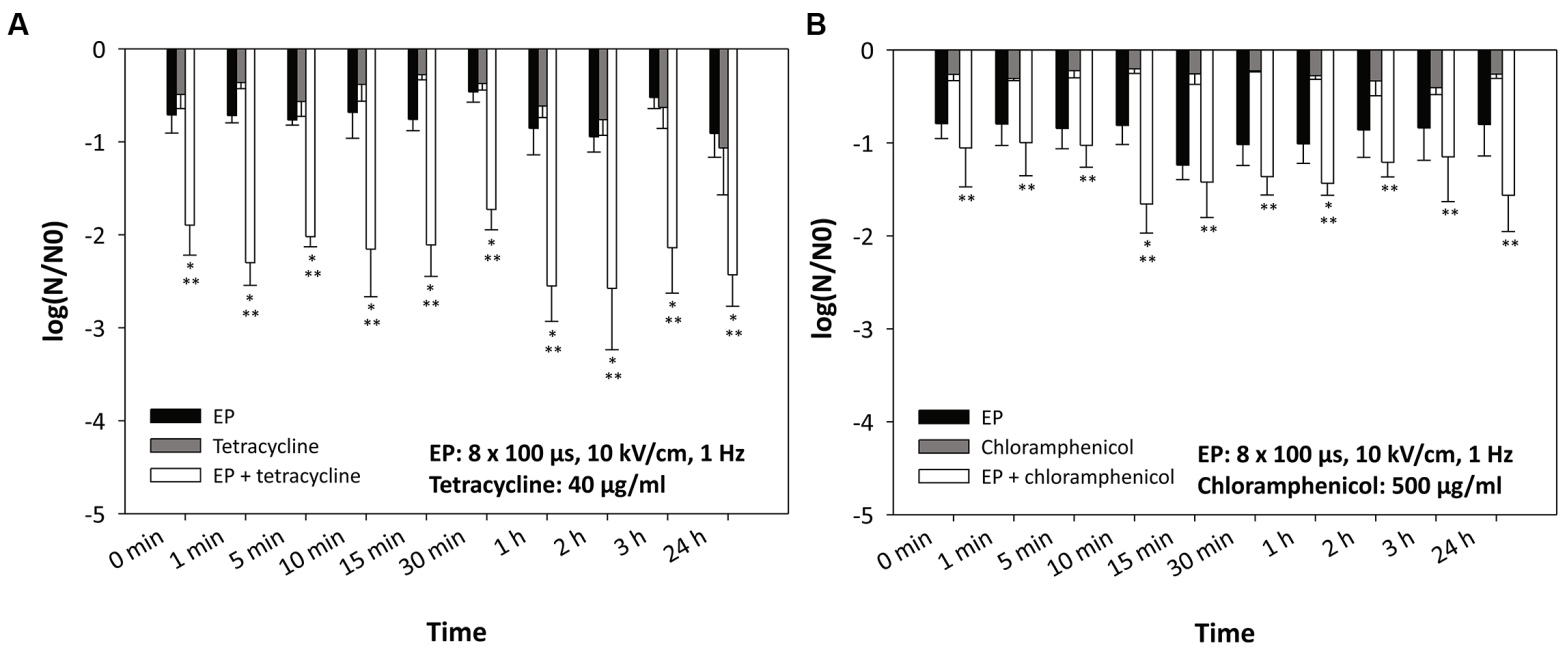
Inactivation of *Escherichia coli* by (A) tetracycline, (B) chloramphenicol, and electroporation – time dynamics. Antibiotics were added at different time points after electroporation. Bacterial cells (*Escherichia coli* ER2420) were grown to early exponential phase and subjected to electroporation (black bars) or treatment with tetracycline or chloramphenicol alone or in combination. Electroporation parameters were: 8 pulses of 100 μs duration, with a repetition rate of 1 Hz and electric field strengths of 10 kV/cm. The concentrations of added tetracycline were 40 μg/mL **(A)** and those of added chloramphenicol were 500 μg/mL **(B)**. Bacterial cells were treated at room temperature (22°C). Bacterial cell count reduction is expressed as log (N/N0), where N is the number of CFU/ml in the treated sample and N0 is the number of CFU/ml in the control sample. An asterisk (*) indicates a statistically significant (*p* < 0.05) difference from electroporation treatment alone, and two asterisks (**) indicate a statistically significant (*p* < 0.05) difference from antibiotic treatment alone. Values represent means (all tests were performed in triplicate), and error bars were calculated using the standard deviation. The abbreviation “EP” stands for “electroporation.”

Figure 4 shows the inactivation of *E. coli* when 40 μg/mL tetracycline (Figure 4A) or 500 μg/mL chloramphenicol (Figure 4B) was added at different times after electroporation. Interestingly, there was no statistically significant difference whether the antibiotic was added immediately after electroporation (0 min) or at different time points after electroporation. A statistically significant effect (compared to treatment with electroporation or antibiotic alone) was observed in combination of electroporation with tetracycline. With chloramphenicol, a statistically significant additional effect was only observed with respect to treatment with chloramphenicol alone.

## 4 Discussion

Bacteria have long been known to be important drivers of both beneficial and harmful processes in natural and artificial ecosystems (Madigan et al., 2021). In the last century, the use of antibiotics has revolutionized modern medicine by providing effective treatment against pathogenic bacteria. However, the overuse, misuse, and improper disposal of antibiotics have resulted in their widespread presence in wastewater. The situation has raised concerns about the potential environmental and human health impacts associated with the release of antibiotic-resistant bacteria into the environment. Therefore, it is crucial to understand the dynamics of bacteria and antibiotics in wastewater in order to develop effective strategies for their management and mitigation (Keen and Fugère, 2017).

Conventional wastewater treatment processes are designed to remove solids and organics from wastewater and reduce levels of harmful bacteria and other pathogens. These processes usually involve several stages, at the end of which disinfection methods such as chlorination or ultraviolet light are employed (Keen and Fugère, 2017). Although these methods are quite reliable, they have been shown not to work under all conditions (e.g., UV disinfection does not work in turbid water) or to result in the formation of undesirable halogenated organics. Electroporation as a non-chemical disinfection method in which no genotoxicity has been observed can therefore be considered a suitable alternative method for reducing the number of harmful bacteria in wastewater (Gusbeth et al., 2009).

It has already been shown that the activity of some enzymes in various liquid foods (e.g., apple juice, soy milk) decreases when subjected to electroporation and that the degree of inactivation depends on the strength of the electric field (Riener et al., 2008a,b, 2009). Therefore, only the antibiotics were subjected to electric pulses with the highest electric field used in our study (20 kV/cm). We did not observe any loss of antibiotic activity in our experiments (see Figure 2). A small, but statistically significant difference was only found at one tetracycline concentration (80 μg/mL). However, since this difference was not observed for all other concentrations, including chloramphenicol, we can conclude that electroporation does not affect the activity of the antibiotics used in this study.

Electroporation renders the cell membrane transiently permeable to molecules which otherwise cannot enter or leave the cell (Kotnik et al., 2010). Therefore, electroporation has been used in combination with cisplatin to treat cisplatin-resistant tumor cells (Cemazar et al., 1998) and in combination with antibiotics to inactivate antibiotic-resistant bacteria (Novickij et al., 2018). To mimic the situation in nature, where the antibiotic is present in the water for a prolonged time, but then its concentration slowly decreases with the inflow of a fresh stream, we first cultured the bacteria together with the antibiotic for 3 h after treatment - but only the samples to which the antibiotic was added. The antibiotic was not added to the other samples (i.e., the untreated bacteria and the bacteria treated only with electroporation), but they were also incubated for 3 h. The bacteria were then cultured in the culture medium without the antibiotic for 24 h to eliminate the influence of the antibiotic’s lingering effect. The same methodology was used for the results presented in Figures 2–4. In our study, we have shown that electroporation in the presence of antibiotics can significantly reduce the number of antibiotic-resistant bacteria in the treated sample. Moreover, we have also shown that the inactivation of bacteria depends on the antibiotic used and its concentration.

Our results (see Figure 3) are in agreement with previous studies (Novickij et al., 2018; Vadlamani et al., 2018; Rubin et al., 2019; Kuyukina et al., 2020; Martens et al., 2020; Vadlamani et al., 2020; Lovsin et al., 2021) in which others have also shown electroporation to enhance antimicrobial activity against bacteria. Moreover, we and others have shown that electroporation in combination with antibiotics is also effective against bacteria that are resistant to these antibiotics (Novickij et al., 2018; Vadlamani et al., 2020). We demonstrated that the combined treatment was more effective at higher electric fields and/or higher antibiotic concentration, which is consistent with previous reports (Novickij et al., 2018; Vadlamani et al., 2018, 2020; Lovsin et al., 2021). The inactivation of chloramphenicol in bacteria requires ATP (VanDrisse and Escalante-Semerena, 2019) and the tetracycline efflux pumps draw energy from various ions (Grossman, 2016). In addition, bacteria require ATP molecules to repair their damaged membrane (e.g., by electroporation). Electroporation (especially when using a higher electric field strength) has been shown to cause a rapid and acute loss of ATP and release of ions (Napotnik et al., 2021). Since ATP and ion loss occurs in the cell after electroporation, this could explain the higher loss of viability of the antibiotic-resistant bacteria used in our study when a combined treatment (electroporation and antibiotics) is performed. In addition, we have shown that inactivation by combined treatment also depends on the bacterial resistance mechanism. The bacterium used in this study pumps tetracycline out of the cell and uses enzymes to render chloramphenicol nonfunctional (see Figure 1). In our study, tetracycline with pumping out resistance mechanism was more effective against *E. coli* compared to chloramphenicol with rendering antibiotic ineffective (see Figure 3).

Both antibiotics have a similar mode of action (inhibition of protein synthesis) (Madigan et al., 2021), both are hydrophilic and penetrate bacteria through general diffusion porins, which are present in large quantities in the outer bacterial membrane (Delcour, 2009), but the bacterial resistance mechanism to both antibiotics is different (see Figure 1). The molecular weight of tetracycline (444 g/mol) is much higher than that of chloramphenicol (323 g/mol), so one would expect that the uptake of chloramphenicol into the bacterium after electroporation would be much higher (due to the permeabilized membrane) and thus the inactivation of the bacterium would be greater. Namely, one of the factors determining molecule transfer through the electropermeabilized membrane is the size of the transferred molecule, i.e., larger molecules have a harder time penetrating the cells after electroporation than smaller ones, as has been shown for nanoparticles (Egloff et al., 2021) and DNA molecules (Sachdev et al., 2020).

**Figure 5:**
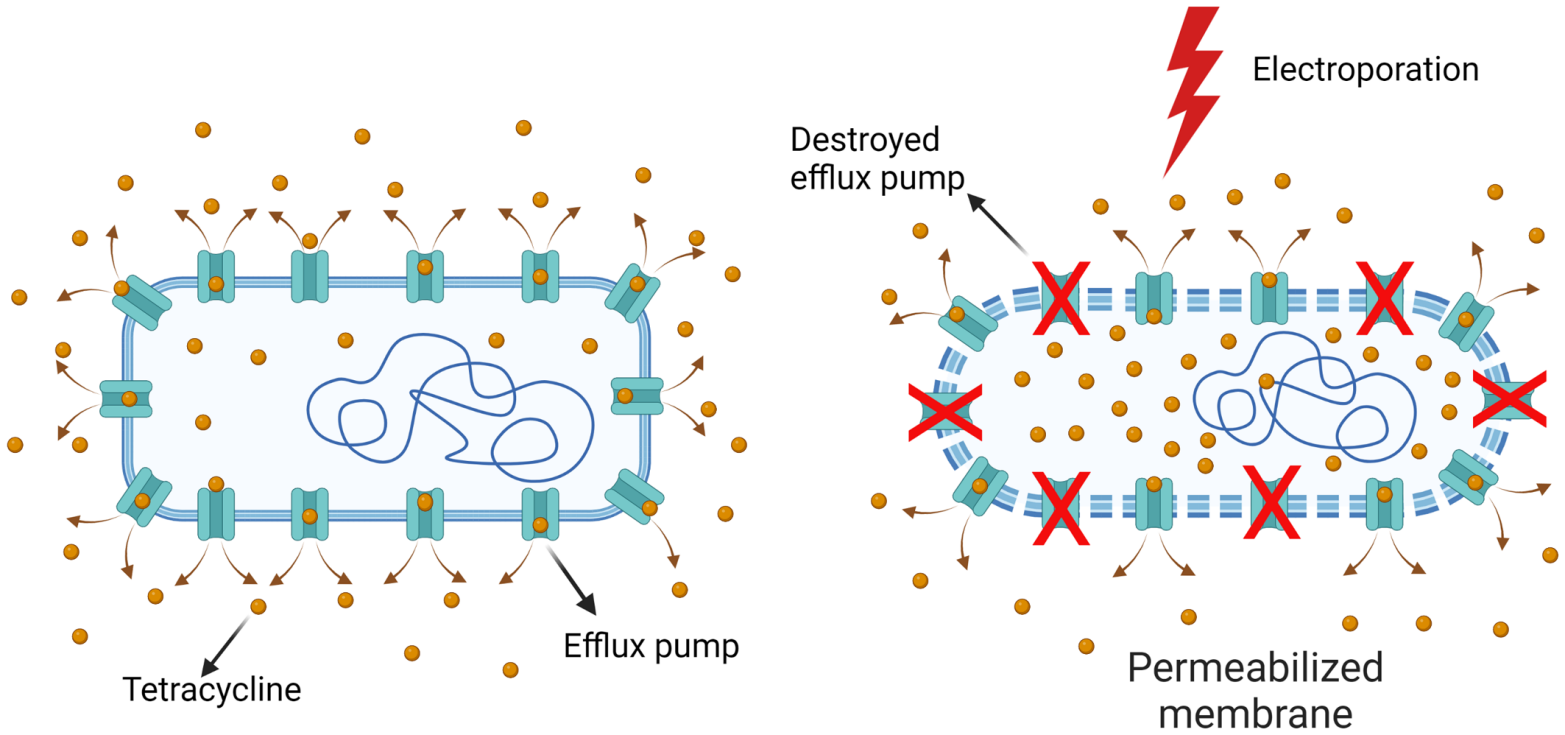
Destruction of the bacterial TetA pump by electroporation. The efflux pumps that enable bacterial resistance are probably denatured to some degree by electroporation, so that the tetracycline concentration inside bacteria is higher than in non-electroporated bacteria. Created with BioRender.com.

One possible explanation could be that the difference may be due to the denaturation of the TetA pump (tetracycline efflux pump in the bacteria used for this study) by electroporation and that the bacterium is no longer able to pump the tetracycline out to the same extent (Figure 5). The role of TetA is to decrease the intracellular presence of tetracycline by pumping it out of the cell at a rate equal to or greater than uptake (Speer et al., 1992; Fernandez and Hancock, 2012). It has been previously shown that electric pulses (which cause permeabilization of the cell membrane) can also cause denaturation of transmembrane proteins (Kotnik et al., 2019). In particular, pulses of shorter duration (shorter than 1 ms) damage the proteins in the plasma membrane of the cell more than the phospholipid bilayer (Huang et al., 2013). Recent molecular dynamics simulations of membrane proteins exposed to electric fields that mimic electroporation conditions have also shown that pores form in some domains of membrane proteins, which renders them dysfunctional (Rems et al., 2020).

Another explanation for the difference in efficacy of the two antibiotics may be that tetracycline, although pumped out of the cell, remains functional and can re-enter the cell through the porous membrane. While the bacterium changes chloramphenicol and the concentration of functional chloramphenicol becomes lower.

In the study by Lovsin et al. (2021), it was reported that other antibiotics in combination with electroporation seem to be more effective than tetracycline, which is not in agreement with our study. One possible explanation is that we used a different bacterial strain (bacteria resistant to tetracycline) and a different initial antibiotic concentration.

By fractioning the electroporation treatment over the period of time, a remarkable increase in bacterial inactivation was shown (Delso et al., 2020). In the study, it was hypothesized that the higher efficacy was due to the restoration of the original membrane permeability. In mammalian cells, it has already been shown that the efficiency of electroporation is increased by fractioning a treatment into more shorter pulses (Pakhomova et al., 2011, 2013; Muratori et al., 2016). It was suggested that electroporation in mammalian cells induces sensitization to subsequent treatments. Indeed, various cellular damages occur after electroporation (Napotnik et al., 2021), such as membrane damage (i.e., pore formation, lipid peroxidation, membrane protein denaturation), influx and efflux of ions and DAMP molecules (Polajzer et al., 2020), production of reactive oxygen species, etc. All of these factors can contribute to the cell being fragile for a prolonged period of time, i.e., more vulnerable to subsequent exposure to various agents. In addition, the antibiotic may float in the wastewater at a later time (e.g., after electroporation treatment). Therefore, to study the sustained effect of electroporation on bacterial cells, we added antibiotics at lower concentrations up to 24 h after electroporation (Figure 4). The results show that the inactivation of bacteria was the same (regardless of the time of antibiotic addition - even 24 h after electroporation) as when the antibiotic was added immediately after electroporation. This could be due to the fact that electroporation has a prolonged effect on cell, as also shown recently (Peng et al., 2023), or there may be other additional factors at play. Namely, some of the bacterial cells release toxins when they are lysed (Granato and Foster, 2020). This happens mainly when bacterial cells are confronted with other bacterial competitors and their task is to eliminate the opponent. In this case, several bacteria that have released toxins are also weakened or damaged, and the so-called mass suicide of cells occurs. Whether this phenomenon also occurs after electroporation remains to be investigated. In addition, an environment that is unfriendly to the bacteria (suboptimal temperature, suboptimal media, etc.) impairs the viability of the bacteria. The incubation time (up to 24 h) in such an environment seems to be another factor that plays a role in our study. Our experiment illustrates the effective use of electroporation for wastewater treatment. Even if the bacteria are treated with electroporation and the antibiotic is not present but is added to the bacteria later (up to 24 h later), this, together with other factors described above (temperature, media, etc.), has a strong effect on the survival of the bacteria.

As a control for the calculation of log (N/N0) in Figure 4, we used bacteria that were not treated (neither by electroporation nor by antibiotics) but were incubated for the same time as the treated bacteria. In these experiments we wanted to observe the long-term effects of the treatment, without taking other factors into account. When the non-incubated control (bacterial count at time 0) is used to calculate log (N/N0), we observe a statistically significant additional effect only for longer incubation times (more than 1 h), which shows that a non-optimal environment for the bacteria also plays a role (as would be the case if bacteria were electroporated in wastewater).

Our results offer ample motivation and directions for further research to better understand the role of electroporation in potentiating antibiotics to inactivate resistant bacteria in the aquatic environment. Although the concentrations of antibiotics in various effluents tend to be low (Lovsin et al., 2021) or they may float in latter, electroporation with appropriate pulse parameters is worth further investigation to reduce the amount of resistant pathogenic species in the aquatic environment. In addition, the use of electroporation to inactivate bacteria in wastewater treatment offers potential advantages such as high efficiency (Rieder et al., 2008; Schoen et al., 2010; Huo et al., 2022), and the absence of harmful disinfection by-products (Gusbeth et al., 2009). However, it is important to consider the wider environmental impact of implementing electroporation in wastewater treatment. The fate of bacterial remains and the potential for the release of genetic material or antibiotic resistance genes from inactivated bacteria into the environment should be considered (Haberl Meglic et al., 2020), as these factors could have an impact on the environment and public health. Indeed, it has been shown that immediate gene transfer from donor to recipient bacteria is possible by electroporation (Kilbane and Bielaga, 1991; Ward and Jarvis, 1991; Kotnik, 2013). To avoid this problem, other complementary downstream processes could therefore be used to remove resistance genes from electroporation-treated water (Mosaka et al., 2022).

Furthermore, the energy requirements and operational aspects of electroporation-based wastewater treatment should be carefully evaluated in order to assess its overall environmental sustainability. Namely, one of the main issues is the high-energy consumption required for the process (Narsetti et al., 2006; Kotnik et al., 2015). Nevertheless, electroporation has already been used for large-scale pasteurization of liquid, semi-solid and solid foods (Barbosa-Cánovas et al., 2022). Although some of the water quality parameters may have a minor (pH, ionic strength and ion type) or major (higher concentration of organic matter) influence on the inactivation of bacteria in water by electroporation (Huo et al., 2018), this technique offers promising possibilities for the inactivation of bacteria in large-scale wastewater treatment. It could also be used as an independent treatment process at critical control points in wastewater treatment, such as on-site treatment of wastewater generated from hospitals or other healthcare facilities.

## 5 Conclusion

In conclusion, antibiotic-resistant bacteria in wastewater present a significant threat. The discharge of untreated wastewater into water bodies can lead to the spread of these bacteria and adversely affect human and animal health. Innovative treatment technologies (such as electroporation) could play a pivotal role in addressing the problem of antibiotic-resistant bacteria in wastewater. Our results presented in this study show that electroporation not only enhances the effect of antibiotics and reduces the number of antibiotic-resistant bacteria, but also has a longer-lasting effect on the bacteria, making them more vulnerable to other factors such as low antibiotic concentration. However, further research is needed to optimize and standardize the conditions for electroporation to ensure its effectiveness.

## Data availability statement

The raw data supporting the conclusions of this article will be made available by the authors, without undue reservation.

## Author contributions

SH: Conceptualization, Data curation, Formal analysis, Investigation, Methodology, Supervision, Validation, Writing – original draft, Writing – review & editing. DS: Conceptualization, Funding acquisition, Supervision, Validation, Writing – review & editing. DM: Methodology, Writing – review & editing.
